# Asymptomatic Aortic Regurgitation: Evolving Imaging Markers and Contemporary Intervention Strategies

**DOI:** 10.3390/jcm15010339

**Published:** 2026-01-02

**Authors:** Chieh-Mei Tsai, Kuan-Yu Lai, Yu-Chien Su, Chi-Han Wu, Casper H. H. Tsai, Shivam Singh, Li-Tan Yang

**Affiliations:** 1 Department of Cardiovascular Medicine, Mayo Clinic, Rochester, MN 55905, USA; tsai.chieh-mei@mayo.edu; 2 Department of Internal Medicine, National Taiwan University Hospital, Taipei 100225, Taiwan; b07401149@ntu.edu.tw (K.-Y.L.);; 3 College of Medicine, National Taiwan University, Taipei 100225, Taiwan; 4 Department of Surgery, National Taiwan University Hospital, Taipei 100225, Taiwan; 5 Department of Internal Medicine, Reading Hospital, West Reading, PA 19611, USA; 6 Division of Cardiology, Department of Internal Medicine, National Taiwan University Hospital, Taipei 100225, Taiwan

**Keywords:** aortic regurgitation, global longitudinal strain, cardiac magnetic resonance, valve repair, Ross procedure, transcatheter aortic valve replacement, multimodal imaging

## Abstract

Asymptomatic aortic regurgitation (AR) has traditionally been managed conservatively until symptom onset or overt left ventricular systolic dysfunction. However, adverse myocardial remodeling—detected by myocardial strain, volumetric cardiac magnetic resonance, and fibrosis imaging—often precedes current guideline thresholds for interventions and may be irreversible. Advances in multimodal imaging now enable earlier risk stratification beyond conventional metrics. In parallel, intervention strategies are evolving, including valve repair, valve-sparing root replacement, Ross procedure, and transcatheter aortic valve replacement in selected high-risk patients. This narrative review summarizes contemporary advances in imaging and intervention for asymptomatic AR, while critically appraising current evidentiary and technical limitations that constrain earlier intervention. The review is based on a narrative synthesis of the contemporary literature, drawing from recent clinical studies, imaging advances, and guideline documents rather than a systematic evidence search.

## 1. Introduction

Aortic regurgitation (AR) is a prevalent valvular disease whose burden is rising as populations age [1]. In community-dwelling adults ≥65 years, mild AR is observed in approximately 7–14%, while moderate-to-severe AR occurs in ~2–5% [2,3,4]. Although many patients remain asymptomatic for prolonged periods, longitudinal cohort data shows that one-fifth of patients with Stage B AR progress to Stage C/D hemodynamic severity—defined by worsening quantitative echocardiographic thresholds for at least moderately severe AR—over a median follow-up of four years, and a substantial subset of these progressors subsequently develop symptoms or meet surgical criteria [5,6]. Delayed surgical referral is associated with irreversible myocardial injury and worse postoperative outcomes, underscoring the limitations of relying on symptoms, left ventricular ejection fraction (LVEF), and linear dimensions alone [7,8,9,10].

Advances in multimodal imaging have reshaped contemporary assessment of AR severity and LV remodeling [11]. Three-dimensional (3D) echocardiography improves quantification in eccentric jets, while cardiac magnetic resonance (CMR) offers highly reproducible volumetric assessment and regurgitant quantification [12,13,14,15,16]. Beyond chamber size, CMR tissue characterization with late gadolinium enhancement (LGE) and indexed extracellular volume (iECV) identifies focal and diffuse myocardial fibrosis associated with adverse outcomes, while myocardial deformation parameters—such as global longitudinal strain (GLS, a measure of LV fiber shortening) and left atrial reservoir strain (reflecting atrial compliance and filling)—detect subclinical dysfunction despite preserved LVEF [17,18].

Therapeutic strategies are also evolving. Aortic valve (AV) repair and valve-sparing root replacement achieve durable outcomes in selected patients, and the Ross procedure remains an option for carefully selected younger individuals, and newer-generation transcatheter devices are expanding treatment options for high-risk patients with native AR [19,20,21]. Together, these advances are shifting management toward earlier and more individualized decision-making. This review synthesizes contemporary data on AR pathophysiology, multimodal imaging, and evolving intervention strategies, with emphasis on the remaining technical, biological, and evidentiary barriers that limit definitive early-intervention paradigms.

## 2. Methods

This article is a narrative review synthesizing contemporary evidence on asymptomatic AR, multimodality imaging, and evolving intervention strategies. We searched PubMed from inception through December 2025 for original studies, meta-analyses, guideline documents, and state-of-the-art reviews relevant to AR pathophysiology, imaging assessment, clinical outcomes, and surgical or transcatheter management. Search terms included combinations of “aortic regurgitation”, “asymptomatic”, “echocardiography”, “strain”, “cardiac magnetic resonance”, “aortic valve repair”, “Ross”, and “transcatheter aortic valve replacement.” We prioritized studies published within the past 10 years, but included seminal older literature when foundational to current understanding. Evidence was selected based on clinical relevance, methodological quality, and contribution to current practice or emerging paradigms; no formal systematic screening, quantitative synthesis, or risk-of-bias assessment was performed.

## 3. Multimodal Imaging in Aortic Regurgitation

Transthoracic echocardiography remains the first-line modality for evaluating AR severity, providing insight into valve anatomy, regurgitation mechanism, and LV remodeling. However, quantification—especially in eccentric jets—can be limited by acoustic dropout, poor alignment, and inaccurate estimation of vena contracta or PISA-based measurements [22]. 3D echocardiography overcomes several of these limitations by enabling direct planimetry of the vena contracta area and volumetric quantification without geometric assumptions [12,13,14]. Comparative studies demonstrate that 3D echocardiography provides more accurate assessment of regurgitant volume and effective regurgitant orifice area, with stronger correlation to CMR, narrower limits of agreement, and superior classification of AR severity—particularly in eccentric or multiple jets—compared with 2D techniques [13,23,24]. Key echocardiographic prognostic thresholds are summarized in Table 1.

Stress echocardiography is also considered for evaluating asymptomatic AR patients, primarily to objectively confirm exercise tolerance and elicit exertional symptoms [35,36]. However, despite its physiologic appeal, stress-derived imaging markers have not been incorporated into guidelines because robust outcome-based evidence is lacking. Existing studies are small and heterogeneous, and no standardized thresholds exist for exercise-induced changes in LV function, volumes, or regurgitant severity that reliably predict prognosis. Consequently, while reduced exercise tricuspid annular plane systolic excursion and absence of contractile reserve have been associated with early surgical referral and symptom development, these findings remain insufficient for guideline integration, and stress testing continues to serve mainly as a functional assessment rather than a quantitative risk-stratification tool [37,38].

CMR is the most reproducible modality for quantifying regurgitant burden and is recommended when echocardiographic findings are inconclusive [39]. Unlike mitral regurgitation (MR), chronic AR imposes both systolic and diastolic volume overload, leading to earlier and more pronounced LV remodeling [40,41]. Although guideline echocardiographic thresholds for severe AR (RV ≥ 60 mL, RF ≥ 50%) are extrapolated from MR [42], AR-specific CMR studies consistently demonstrate prognostic thresholds at lower values. Reported cutoffs vary (RV 38–47 mL; RF 32–43%) because of differences in phase-contrast measurement location, the use of direct versus indirect flow-quantification techniques, and heterogeneity in clinical endpoints across studies [11,15,16,43,44]. The CMR-based prognostic thresholds are summarized in Table 2. Notably, the upper end of these ranges was validated in a recent large multicenter cohort of asymptomatic patients with moderate-severe AR and preserved LVEF [15]. These data-driven thresholds highlight the potential for earlier risk identification and referral. Cardiac computed tomography may assist in select cases by delineating aortic root anatomy and regurgitant orifice geometry but is limited by flow artifacts and suboptimal leaflet definition [11].

## 4. Early Detection of LV Dysfunction

Imaging plays a central role in detecting subclinical LV dysfunction and refining the timing of intervention in chronic AR. Traditional referral thresholds—LVEF < 55% or LV end-systolic diameter (LVESD) > 50 mm—often indicate late-stage disease [9,45]. Accumulating evidence supports the use of indexed LV dimensions and volumes as earlier, more sensitive markers of adverse remodeling [7,25,28,29,30]. Yang et al. first demonstrated that the LV end-systolic dimension index (LVESDi) > 20 mm/m^2^ was associated with all-cause mortality, superior to LVEF and absolute LV dimensions [7]. As summarized in Table 1, this threshold has since been reproduced across large European and Asian multicenter cohorts [25,29], where risk begins to rise at LVESDi values in the 20–22 mm/m^2^ range. Multicenter CMR studies summarized in Table 2 further reinforce the prognostic superiority of volumetric measures, particularly LVESVi ≥ 45 mL/m^2^, which remain strong independent predictors across modalities [15,16]. Current guidelines rely on absolute LV cutoffs and do not define trajectory-based triggers; however, progressive LV enlargement below thresholds and rapid remodeling warrant closer surveillance [16,29,45], while no condition-specific LV thresholds are established for bicuspid valve disease, connective tissue disorders, chronic kidney disease, hypertension, or athletic hearts—necessitating individualized interpretation. Sex- and body size–specific differences in LV remodeling also represent an important source of risk misclassification in chronic AR. Women and smaller-bodied patients, including many Asian populations, exhibit blunted LV dilatation despite comparable regurgitant burden, lower volumetric thresholds for adverse outcomes, and sex-specific strain reference values, limiting the applicability of uniform cutoffs [46,47,48,49,50,51,52].

While numerous studies have identified individual echocardiographic markers associated with outcomes (Table 1), long-term data also highlight the clinical consequences of delayed intervention. In the prospective study by Tornos et al., adherence to guideline-based timing of surgery was associated with markedly improved 15-year survival, underscoring the importance of avoiding surgery only after the onset of severe symptoms or marked LV dilation [27]. More recently, the Aortic Valve Insufficiency and Ascending Aorta Aneurysm International Registry (AVIATOR) registry demonstrated that surgery triggered solely by conventional class I indications (symptomatic heart failure, LVEF ≤ 50% or LVESD > 50 mm/LVESDi > 25 mm/m^2^) was associated with reduced long-term survival after surgery, whereas earlier intervention—at LVESDi 20–25 mm/m^2^ or LVEF 50–55%—was not associated with diminished survival [26]. These observations inform modern recommendations. The 2020 American College of Cardiology/American Heart Association (ACC/AHA) guideline maintains LVEF ≤ 55% as a Class I indication for surgery in asymptomatic severe AR (Stage C2) [45]. For patients with preserved LVEF (>55%) but severe LV dilation (LVESD > 50 mm or LVESDi > 25 mm/m^2^) surgery is reasonable (Class IIa) [45]. In asymptomatic patients with normal LV systolic function (LVEF > 55%; Stage C1) and low surgical risk, surgery may be considered (Class IIb) when serial studies show a progressive decline in LVEF into the low-normal range (55–60%) or a progressive increase in LV size into the severe range (left ventricular end-diastolic diameter [LVEDD] > 65 mm) [45]. The 2025 European Society of Cardiology (ESC) and the European Association for Cardio-Thoracic Surgery (EACTS) guideline further refines surgical triggers by lowering the indexed linear cutoff to LVESDi > 22 mm/m^2^ (LOE B) and introducing a volumetric trigger of LVESVi > 45 mL/m^2^ (LOE B), while maintaining the LVEF cutoff of ≤55% [15,53]. Traditional diameter-based progression criteria (e.g., LVEDD > 65 mm or serial enlargement) remain LOE C because of limited prospective validation. Despite these updated dimensional and volumetric thresholds, the optimal timing of intervention in asymptomatic severe AR with preserved LVEF remains uncertain, as accumulating evidence suggests that myocardial damage may occur before current Class I indications are reached, underscoring the need for individualized assessment beyond guideline cutoffs [45,53].

Although CMR provides superior reproducibility for volumetric assessment and AR quantification, echocardiography remains the first-line imaging modality due to its accessibility, cost-effectiveness, and ability to characterize valve mechanism, regurgitant severity, and serial remodeling [15,16,50,51]. For centers without routine CMR availability, guideline-directed echocardiographic thresholds—LVEF ≤ 55%, LVESD > 50 mm or LVESDi > 25 mm/m^2^, and progressive LV dilation—continue to guide surgical decision-making, with CMR reserved for cases in which echocardiographic findings are borderline, discordant, or inconclusive [54,55].

CMR enables myocardial tissue characterization that adds prognostic information beyond LV size and function. LGE, reflecting irreversible focal myocardial scar, identifies a high-risk AR phenotype associated with increased mortality, while iECV, a marker of diffuse interstitial fibrosis, correlates with regurgitant severity, LV remodeling, and adverse outcomes including death and need for AVR (Table 2) [17,18,56,57]. However, CMR-derived measurements remain subject to technical and operator-dependent variability, and inter-center heterogeneity in iECV quantification remains an important limitation [56,57,58]. Accurate interpretation of LGE and fibrosis patterns also depends on reader expertise.

Myocardial strain imaging provides a sensitive measure of myocardial deformation and detects subclinical dysfunction before changes in chamber size or ejection fraction become apparent. LV-GLS is the most widely used clinical strain parameter and serves as a more sensitive marker of early systolic dysfunction than LVEF in valvular disease, heart failure with preserved EF, and cardiotoxicity monitoring [59]. In asymptomatic chronic AR, impaired LV-GLS (typically worse than −15% to −19%) predicts symptom progression and mortality despite preserved LVEF [31,32,60,61]. Integrating LV-GLS with structural markers amplifies prognostic discrimination: the addition of LV-GLS to a clinical risk model improves long-term mortality prediction [31], and an LV-GLS < 15% combined with LVESVi ≥ 45 mL/m^2^ increased mortality risk fourfold, while pairing GLS < 15% with LVESDi > 20 mm/m^2^ increased mortality risk threefold [32]. Left atrial (LA) strain provides complementary physiologic information by reflecting LV diastolic function and filling pressure [59]. In ≥moderate-severe asymptomatic AR, reduced LA reservoir and contractile strain independently predict outcomes, and offer incremental risk stratification beyond LV-GLS [34]. Multicenter data demonstrate sex- and age-independent LA remodeling, with LA volume index ≥37 mL/m^2^ and LA reservoir strain ≤35% identifying higher-risk patients who derive surgical benefit [33].

Despite these strengths, discordance between LV dimensions/volumes, strain, and CMR-derived markers is common. In such cases, expert consensus prioritizes symptoms and LVEF as the dominant triggers for intervention, with CMR volumetric assessment serving as the preferred arbiter when echocardiographic and strain findings disagree [11,54]. Although multiparametric frameworks consistently outperform single-parameter strategies for outcome prediction [28,32,53,60,61], no guideline-endorsed or prospectively validated multimodal algorithm currently exists to direct intervention timing in asymptomatic AR.

However, strain measurements remain constrained by technical variability and limited standardization. GLS exhibits intra-observer variability and vendor dependence, and serial assessment should ideally be performed using the same platform with emphasis on longitudinal change [62,63,64]. LA strain is feasible but highly software dependent, with systematic inter-vendor bias despite improved reproducibility using dedicated tracking tools [65,66,67].

Complementing these imaging markers, machine learning approaches have also been explored. Anand et al. have developed machine learning models that integrate clinical and with indexed LV volumes, contributing to an overall concordance index of 0.87 for survival prediction in severe AR [68]. However, these approaches remain exploratory: most models are developed from retrospective datasets, are vulnerable to overfitting and limited generalizability, and lack prospective and external validation demonstrating improvement in clinical outcomes, so machine learning–derived scores should currently be regarded as hypothesis-generating adjuncts rather than stand-alone triggers for intervention [69,70].

In aggregate, these data support a multimodal imaging framework in chronic AR—integrating echocardiography, CMR, and strain imaging—to refine risk stratification, while recognizing that prospective validation is still required and that technical, operator-, software-, and center-dependent constraints remain important real-world barriers to uniform implementation.

## 5. Surgical and Percutaneous Interventions

Despite advances in imaging and risk stratification, the management of chronic AR remains anchored in timely intervention. The 2020 ACC/AHA guidelines clarify that there is no evidence to support vasodilator therapy for chronic asymptomatic AR in the absence of systemic hypertension, reinforcing that no medical therapy can halt or reverse disease progression in these patients [45]. At present, there are no active disease-modifying pharmacologic trials for chronic AR, and contemporary research has focused primarily on post–aortic valve replacement secondary prevention rather than modification of the natural history of native valve regurgitation. This therapeutic gap underscores the primacy of surgical and transcatheter management.

Surgical correction remains the cornerstone of management for severe AR, with long-term outcomes highly dependent on both timing and type of intervention [71]. Valve replacement provides predictable durability, whereas repair and valve-sparing strategies—when feasible—offer the advantage of avoiding prosthetic-related complications at the cost of potentially higher reintervention risk. Newer transcatheter approaches provide alternatives for select high-risk patients. The following sections review the indications, outcomes, and evolving landscape of AR interventions. A comparative summary of the major surgical and transcatheter intervention strategies is provided in Table 3.

## 6. Aortic Valve Repair and Valve-Sparing Aortic Root Replacement

AV repair has emerged as a compelling alternative to valve replacement in select patients with chronic AR, particularly younger individuals and those with pliable, non-calcified tricuspid or bicuspid valves and repairable mechanisms such as cusp prolapse or annular dilation [80,81,82]. Compared with valve replacement, repair avoids prosthesis-related complications including anticoagulation, thromboembolism, and structural valve deterioration [81]. Contemporary techniques include cusp plication, geometric height correction, patch repair, and valve-sparing aortic root replacement (VSARR), frequently supported by standardized annuloplasty [80,83,84,85].

However, repair feasibility and durability remain highly operator- and center-dependent, reflecting a substantial learning curve and limiting broad generalizability [80,85]. Population-based data indicate that most patients undergoing surgery for AR still receive valve replacement, whereas experienced referral centers report repair rates approaching 50–60% in carefully selected tricuspid valves [86,87]. Outcomes from expert centers demonstrate excellent survival and freedom from reoperation exceeding 85–90% at 5 to 10 years, with multicenter registries extending these findings to more complex anatomies, including bicuspid valves and root dilation, albeit with continued dependence on institutional expertise [19,75,80,88,89,90]. Predictors of reduced durability include preoperative severe AR, advanced LV dilation, and patch use [19,75].

Importantly, no randomized trials directly compare AV repair with SAVR in chronic AR, and existing evidence is derived almost exclusively from observational series at specialized centers [45,85,88]. Reflecting this evolving but limited evidence base, the 2025 ESC/EACTS guidelines upgraded AV repair to a Class IIa (Level B) recommendation for selected patients at experienced centers when durable results are anticipated [53].

## 7. Ross Procedure: Pulmonary Autograft Aortic Replacement

The Ross procedure replaces the diseased aortic valve with the native pulmonary valve and avoid lifelong anticoagulation, offering physiologic hemodynamics and survival advantages in carefully selected younger patients [91,92,93,94,95]. Comparative studies and network meta-analyses consistently show superior long-term survival, lower rates of stroke and endocarditis, and improved quality of life compared with mechanical or bioprosthetic AVR [94,95]. Although increasingly applied in chronic AR, durability is lower than in AS due to higher risk of late autograft dilatation and reoperation, particularly in patients with large annuli or bicuspid/unicuspid valve morphologies [73,74,96,97]. Recent technical refinements, including autograft reinforcement and tailored root stabilization, have mitigated these risks and expanded the role of the Ross procedure within high-volume specialized centers [20,74,98,99,100]. However, published Ross outcomes are subject to strong selection and referral bias, with limited long-term durability data beyond expert centers—particularly in AR, where late autograft failure remains a dominant concern [45,101,102].

## 8. Transcatheter Aortic Valve Replacement

Although surgical aortic valve replacement (SAVR) remains the standard of care for symptomatic AR or LV dysfunction, many patients are not referred for surgery because of advanced age, frailty, or major comorbidities, making transcatheter aortic valve replacement (TAVR) an alternative in carefully selected individuals, particularly those at prohibitive or high surgical risk [77,78,103,104,105,106]. Early experience with TAVR for native AR relied largely on off-label use of devices designed for AS. Non-calcified annuli, annular and root dilation, and limited fluoroscopic landmarks contributed to high rates of valve embolization or migration (12–16%), residual moderate or greater paravalvular regurgitation (9–11%), and pacemaker implantation, each independently associated with adverse outcomes [21,77,78,105,106].

Dedicated AR devices have improved procedural success. The ALIGN-AR (Aortic Valve Implantation for Unoperable Patients With Aortic Regurgitation) pivotal trial—a prospective, multicenter, single-arm study—evaluated the JenaValve Trilogy™ system in patients with high-risk symptomatic native AR and demonstrated high device success with acceptable early safety outcomes [78]. However, nearly half of screened patients were excluded in ALIGN-AR because of unfavorable anatomic or clinical characteristics, and other contemporary observational cohorts remain highly selected and subject to substantial referral, anatomic, and frailty-related selection bias, underscoring the limited generalizability of current evidence base [77,78,105]. Meta-analyses confirm higher procedural success and lower early mortality with dedicated AR devices compared with off-label valves; however pacemaker implantation remains frequent (21–24%), and long-term durability, leaflet thrombosis risk, and the consequences of residual AR remain incompletely defined—particularly as TAVR expands toward younger and lower-risk populations [21,48,76,77,78,79,105,106,107,108].

Transcatheter therapy is now being explored in two high-need populations. The JenaValve ALIGN-AR LVAD Registry (JENA-VAD) is a prospective, multicenter registry nested within the ALIGN-AR program evaluating the Trilogy™ system in patients with continuous-flow left ventricular assist devices and clinically significant AR—a population historically excluded from surgical and transcatheter trials and associated with poor outcomes (ClinicalTrials.gov identifier: NCT06594705). In parallel, the Aortic Regurgitation Trial Investigating Surgery Versus Trilogy™ (ARTIST) is the first randomized controlled trial comparing TAVR with SAVR in patients with clinically significant native AR (ClinicalTrials.gov identifier: NCT06608823). Designed as a large, international non-inferiority trial with long-term follow-up, ARTIST has the potential to define the comparative effectiveness of transcatheter versus surgical therapy in non-prohibitive-risk patients, although enrollment is ongoing and results are not yet available.

Reflecting these evolving but limited data, current guidelines remain conservative. The 2025 ESC/EACTS guidelines introduce a Class IIb recommendation for TAVR in selected inoperable AR patients at experienced centers using dedicated devices, whereas the 2020 ACC/AHA guidelines maintain a Class III (harm) recommendation against TAVR in operable isolated AR [45,53,78]. Pending results from ARTIST and longer-term durability data, TAVR for native AR should remain restricted to carefully selected high-risk or inoperable patients within experienced centers.

## 9. Conclusions

Multimodal imaging has redefined risk stratification in chronic AR by enabling earlier detection of adverse LV remodeling through indexed dimensions, volumetric CMR parameters, myocardial strain, and atrial mechanics, now partially reflected in updated guideline thresholds. However, most available data derive from retrospective or single-center cohorts involving highly selected populations, which limits generalizability and underscores the need for prospective multicenter validation. The absence of randomized CMR- or strain-guided early intervention trials and limited long-term durability data for transcatheter platforms remain critical barriers to more aggressive guideline adoption, underscoring the need for prospective early-intervention studies [109].

## Figures and Tables

**Table 1:** Transthoracic Echocardiographic Prognostic Markers in Chronic Aortic Regurgitation.

Marker	Prognostic Cut-Off(s)	Associated Outcome(s)	Study	Year
LV Systolic Function				
LVEF	≤52%	Cardiovascular mortality	Yang et al. [25]	2023
	≤53%	All-cause mortality		
	<55%	All-cause mortality, cardiovascular events	de Meesteret al. [9]	2019
	50–55%	No increase in all-cause mortality with early AVS	Hanet et al. [26]	2024
LV Linear Dimension				
LVEDD	≥65 and 70 mm	No increase in all-cause mortality after AVS	de Meester et al. [9]	2019
	>65 mm	No increase in all-cause mortality after AVS	Hanet et al. [26]	2024
LVESD	>50 mm	All-cause mortality after AVS	Hanet et al. [26]	2024
	>55 mm *	All-cause mortality, cardiac mortality, or death from heart failure or sudden death *	Tornos et al. [27]	2006
LVESDi	≥20 mm/m^2^	All-cause mortality	Mentias et al. [28]	2016
	>20, 20–25, ≥25 mm/m^2^	All-cause mortality	Yang et al. [7]	2019
	>20, >22 mm/m^2^	All-cause mortality, cardiovascular events	de Meester [9]	2019
	≥20 mm/m^2^	All-cause mortality	Lopez Santi et al. [29]	2025
	≥22 mm/m^2^	All-cause mortality	Yang et al. [25]	2023
	≥23 mm/m^2^	Cardiovascular mortality		
	>25 mm/m^2^	All-cause mortality after AVS	Hanet et al. [26]	2024
LV Volumetric Measure				
LVESVi	>45 mL/m^2^	All-cause mortality under medical surveillance	Yang et al. [30]	2021
	≥46 mL/m^2^	Cardiovascular mortality	Yang et al. [25]	2023
	≥48 mL/m^2^	All-cause mortality		
	≥45 mL/m^2^	All-cause mortality	Lopez Santi et al. [29]	2025
Myocardial and Atrial Strain				
LVGLS	>−19%	All-cause mortality	Alashi et al. [31]	2020
	>−15%	All-cause mortality	Yang et al. [32]	2022
LASr	≤35%	HF hospitalization, urgent AVS, or all-cause mortality	Akintoye et al. [33]	2024
	<40%	All-cause mortality under medical surveillance	Lai et al. [34]	2024
LASct	<20%			

* In the study by Tornos et al., these markers were not assessed as independent predictors. Instead, they were used as part of a composite set of criteria (Presence of LVEF < 45%, LVESD > 55 mm or New York Heart Association Class III-IV symptoms) to define a “too late” surgery group. ARF = aortic regurgitant fraction; AVS = Aortic valve surgery; LVGLS = left ventricular global longitudinal strain; HF = heart failure; LASct = left atrial contractile strain; LASr = left atrial reservoir strain; LV = left ventricular; LVEDD = left ventricular end-diastolic diameter; LVEF = left ventricular ejection fraction; LVESD = left ventricular end-systolic diameter; LVESDi = left ventricular end-systolic diameter index; LVESVi = left ventricular end-systolic volume index.

**Table 2:** Cardiac Magnetic Resonance Prognostic Markers in Chronic Aortic Regurgitation.

Marker	Prognostic Cut-Off(s)	Associated Outcome(s)	Study	Year
Regurgitant Severity				
ARF	≥32%	All-cause mortality, HF hospitalization, or progression of NYHA under medical surveillance	Hashimoto et al. [16]	2022
	>35%	Need for AVR or repair	Vejpongsa et al. [43]	2022
	≥43%	Composite adverse outcome *	Malahfji et al. [15]	2023
ARV	>38 mL	Need for AVR or repair	Vejpongsa et al. [43]	2022
	≥47 mL	Composite adverse outcome *	Malahfji et al. [15]	2023
LV Linear Dimension				
LVESD	≥40 mm	Composite adverse outcome *	Malahfji et al. [15]	2023
LVESDi	≥20 mm/m^2^	Composite adverse outcome *	Malahfji et al. [15]	2023
LV Volumetric Measure				
LVESVi	≥43 mL/m^2^	Composite adverse outcome *	Malahfji et al. [15]	2023
	>45 mL/m^2^	All-cause mortality, HF hospitalization, or progression of NYHA functional class under medical surveillance	Hashimoto et al. [16]	2022
LVEDVi	≥109 mL/m^2^	Composite adverse outcome *	Malahfji et al. [15]	2023
Tissue Characterization				
LGE	Presence	All-cause mortality	Malahfji et al. [17]	2020
iECV	≥24 mL/m^2^	All-cause mortality or the need for AVR	Senapati et al. [18]	2021

* Composite adverse outcome defined as development of symptoms, decline in LVEF to <50%, A fulfillment of guideline-recommended LV dilation thresholds for AVR referral, or all-cause mortality during medical surveillance. AVR = aortic valve replacement; ARF = aortic regurgitant fraction; ARV = aortic regurgitant volume; HF = heart failure; iECV = indexed extracellular volume; LGE = late gadolinium enhancement; LV = left ventricular; LVEDVi = left ventricular end-diastolic volume index; LVESD = left ventricular end-systolic diameter; LVESDi = left ventricular end-systolic diameter index; LVESVi = left ventricular end-systolic volume index; NYHA = New York Heart Association (functional class).

**Table 3:** Landmark Outcomes of Surgical and Transcatheter Interventions for Chronic Aortic Regurgitation.

Study Type	N	Population/Technique	Primary Outcome	Follow-Up	Reference
SAVR					
Single-center cohort	539	Severe AR with reduced LVEF	Operative mortality 5.5%; 10-yr survival 63%	10 years	Chaliki et al., 2022 [72]
Ross Procedure					
Single-center cohort	129	Pure AR, bicuspid AV	20-yr survival 95%; 20-yr freedom from autograft reoperation 85%	20 years	Poh et al., 2018 [73]
Single-center cohort	292	AR vs. AS	15-yr survival 86% vs. 93%; 10-yr freedom from reoperation 80% vs. 95%	15 years	Abeln et al., 2025 [74]
Valve Repair/VSARR					
Single-center cohort	127	AV repair, tricuspid AV, isolated AR	10-yr survival 81%; 10-yr freedom from reoperation 80%	10 years	Tamer et al., 2023 [75]
Multicenter database	7126	Various AV repair/sparing techniques	10-yr reintervention 9–20%	10 years	Zito et al., 2025 [19]
TAVR (Off-label)					
Multicenter registry	331	Pure native AR (early vs. new-generation devices)	1-yr mortality 16%; device success 61% vs. 81%; PVL ≥ moderate 19% vs. 4%	1 year	Yoon et al., 2017 [76]
Multicenter registry	201	Pure native AR (self vs. balloon-expandable)	Device success 76% vs. 84%; valve embolization/migration 15% vs. 16%; PVL ≥ moderate 9% vs. 10%	1 year	Poletti et al., 2023 [77]
TAVR (Dedicated)					
Prospective trial	180	High-risk symptomatic AR	1-yr mortality 8%; technical success 95%; PPI 24%	1 year	Vahl et al., 2024 [78]
Prospective trial	700	High-risk symptomatic AR	2-yr mortality 13%; technical success 95%; PPI 22%	2 years	Makkar et al., 2025 [79]

AR = aortic regurgitation; AV = aortic valve; AS = aortic stenosis; LVEF = left ventricular ejection fraction; PPI = permanent pacemaker implantation; PVL = paravalvular leak.

## Data Availability

No new data were created or analyzed in this study.

## Abbreviations

AR	Aortic regurgitation
AVR	Aortic valve replacement
CMR	Cardiac magnetic resonance
GLS	Global longitudinal strain
iECV	Indexed extracellular volume
LGE	Late gadolinium enhancement
LV	Left ventricle/left ventricular
LVEF	Left ventricular ejection fraction
LVESVi	Left ventricular end-systolic volume index
TAVR	Transcatheter aortic valve replacement
